# Development and Validation of a Real-Time PCR for Detection of Pathogenic *Leptospira* Species in Clinical Materials

**DOI:** 10.1371/journal.pone.0007093

**Published:** 2009-09-18

**Authors:** Ahmed Ahmed, Mirjam F. M. Engelberts, Kimberly R. Boer, Niyaz Ahmed, Rudy A. Hartskeerl

**Affiliations:** 1 WHO/FAO/OIE and National Collaborating Centre for Reference and Research on Leptospirosis and Section of Epidemiology, Department of Biomedical Research, Royal Tropical Institute (KIT), Amsterdam, The Netherlands; 2 Pathogen Biology Laboratory, School of Life Sciences, University of Hyderabad, Hyderabad, India; Charité-Universitätsmedizin Berlin, Germany

## Abstract

Available serological diagnostics do not allow the confirmation of clinically suspected leptospirosis at the early acute phase of illness. Several conventional and real-time PCRs for the early diagnosis of leptospirosis have been described but these have been incompletely evaluated. We developed a SYBR Green-based real-time PCR targeting *secY* and validated it according to international guidelines. To determine the analytical specificity, DNA from 56 *Leptospira* strains belonging to pathogenic, non-pathogenic and intermediate *Leptospira* spp. as well as 46 other micro-organisms was included in this study. All the pathogenic *Leptospira* gave a positive reaction. We found no cross-reaction with saprophytic *Leptospira* and other micro-organisms, implying a high analytical specificity. The analytical sensitivity of the PCR was one copy per reaction from cultured homologous strain M 20 and 1.2 and 1.5 copy for heterologous strains 1342 K and Sarmin, respectively. In spiked serum & blood and kidney tissue the sensitivity was 10 and 20 copies for M 20, 15 and 30 copies for 1342 K and 30 and 50 copies for Sarmin. To determine the diagnostic sensitivity (DSe) and specificity (DSp), clinical blood samples from 26 laboratory-confirmed and 107 negative patients suspected of leptospirosis were enrolled as a prospective consecutive cohort. Based on culture as the gold standard, we found a DSe and DSp of 100% and 93%, respectively. All eight PCR positive samples that had a negative culture seroconverted later on, implying a higher actual DSp. When using culture and serology as the gold standard, the DSe was lower (89%) while the DSp was higher (100%). DSe was 100% in samples collected within the first – for treatment important - 4 days after onset of the illness. Reproducibility and repeatability of the assay, determined by blind testing kidney samples from 20 confirmed positive and 20 negative rodents both appeared 100%. In conclusion we have described for the first time the development of a robust SYBR Green real-time PCR for the detection of pathogenic *Leptospira* combined with a detailed assessment of its clinical accuracy, thus providing a method for the early diagnosis of leptospirosis with a well-defined satisfactory performance.

## Introduction

Leptospirosis is a zoonotic disease caused by pathogenic bacteria of the genus *Leptospira*, which are transmitted directly or indirectly from animals to humans. Leptospirosis occurs worldwide but is most common in tropical and subtropical areas with high rainfall [1], [2]. Globally, an estimated number of 500,000 severe cases occur annually with case fatality rates ranging from 3% to 70%, depending on the clinical manifestations [2], [3]. Although the exact number of mild cases is unknown, it is probable that the burden exceeds that of severe leptospirosis. Preliminary results indicate that the present reported incidence of severe leptospirosis may present a significant underestimation of the actual cases, making leptospirosis potentially one of the major neglected infectious diseases. This underestimation is in part due to the non-characteristic manifestations of the disease; leptospirosis is often confused with other diseases that are endemic and epidemic in similar environmental and climatologic conditions such as dengue, rickettsiosis, enteric fevers and malaria. Thus clinical diagnosis alone is not adequate in most cases and laboratory confirmation is essential.

It is important to note, that in contrast to many of the resembling diseases (e.g. dengue), leptospirosis can easily be treated with antibiotics. This is provided that the diagnosis is confirmed before the 5th day after disease onset, when treatment with antibiotics is most effective [2], [4], [5]. One of the most important current diagnostic tests, the microscopic agglutination test (MAT), which is often used as the gold standard, is based on serology and can only confirm the disease at a later acute phase because anti-*Leptospira* antibodies generally become detectable only 5 to 7 days after onset of illness. Thus to enable starting treatment at the most effective time point, the availability of an accurate diagnostic test that is reliable in the early acute phase of the disease, is essential for the most optimal treatment.

The Polymerase Chain Reaction (PCR) technique, which can detect the DNA of pathogenic leptospires present in the blood of the patient in the first 5 to 10 days, may be a promising tool for such early laboratory diagnosis.

PCR-based diagnostics have been effectively developed for a wide range of microbes. Due to its high sensitivity, specificity and speed of amplification, the PCR has been shown to be extremely useful for detecting and identifying organisms in instances where existing culture techniques have failed or have been inadequate [6], [7]. In the last two decades, several conventional PCRs for the diagnosis of leptospirosis have been described, using a variety of target genes, including *rrs* [8], *flab* [9] and *ompL1* [10]. One of these PCRs used two primers pairs, i.e. primer pair G1/G2 amplifying DNA from all pathogenic *Leptospira* spp. except *L. kirschneri* and *L. kirschneri*-specific primer pair B64I/B64II [11]. When combined with Southern blotting using specific probes, this PCR was highly specific for pathogenic *Leptospira* and had a detection threshold of about 10 bacteria when applied to a variety of clinical samples [12], making it useful for diagnostic purposes [13]. Primers G1 and G2 target the *secY* gene [14].

Recently, a number of real-time PCRs were introduced as a rapid and sensitive tool for leptospires detection, reducing the risk of false positive results by carry-over contamination. PCRs targeting the *ligA, B* genes [15], *rrs* gene [16], *gyrB* gene [17], the conserved hypothetical protein coding locus LA0322 in *L. interrogans* serovar Lai [18] and *lipL32* [19], [20] are claimed to be specific for pathogenic *Leptospira* and therefore appropriate for diagnostic purposes. However, considering the low sequence drift in *rrs* [21]–[22], it is questionable whether this gene presents an optimal target to discriminate between pathogenic and saprophytic leptospires. Besides, it is not always clear whether the amplicons are well suited for species or strain discrimination, either by sequencing following conventional amplification or by determining characteristic melting temperatures (Tm).

The gene *secY* encoding preprotein translocase for *Leptospira* is located within the *S10-spc-α* locus containing genes for ribosomal proteins [14], [23]. *secY* is a house keeping gene that consists of alternating conserved and variable regions, making it suitable to deduce primers that generate amplicons with sufficient sequence heterogeneity to enable phylogenetic interpretation for *Leptospira* [23]–[26].

The primary aim of the study is to develop a real-time PCR that specifically detects pathogenic *Leptospira* and to determine its diagnostic accuracy, including parameters such as sensitivity, specificity and reproducibility. Secondly, this PCR should generate an amplicon with sequence variability suitable for phylogenic assessment for molecular epidemiological purposes.

## Results

### Design of the real-time PCR

Optimal conditions for the real-time PCR were determined by performing reactions under various conditions using 5.1 10^−12^ g DNA, equivalent to 1000 genome copies, from *Leptospira* strain M 20. Optimal conditions are mentioned as PCR profile in the Material and Methods section. The optimal amount of internal amplification control (IAC) was estimated as 0.87 10^−15^ g per reaction volume, the sequences of the generated amplicons had a similar high phylogenic potential as found before for G1/G2 generated amplicons (Fig. S1).

### Analytical specificity and sensitivity of the assay

Primer set SecYIVF/SecYIV specifically amplified DNA from pathogenic *Leptospira* (Table 1). Intermediate and doubtful species gave a negative result, except for *L. inadai*, serovar Kaup, strain LT 64–68 that gave a positive PCR result and *L. inadai*, serovar Lincang, strain L14 and *L. meyeri*, serovar Perameles, strain Bandicoot 343 that gave ambiguous results. A BLAST search in GenBank did not reveal disturbing sequence identities between the primers and *secY* of other micro-organisms. Consistently, we found no cross-reaction with DNA from 46 other micro-organisms, implying a high analytical specificity of the test (Table 2).

**Table 1:** *Leptospira* strains used in this study.

No.	Serovar	Serogroup	Strain	Species	Status	Result	Reference
1	Hardjo	Sejroe	Hardjoprajitno	*L. interrogans*	Pathogenic	+	Brenner et al., 1999
2	Hebdomadis	Hebdomadis	Hebdomadis	*L. interrogans*	Pathogenic	+	Brenner et al., 1999
3	Canicola	Canicola	Hond Utrecht IV	*L. interrogans*	Pathogenic	+	Brenner et al., 1999
4	Lai	Icterohaemorrhagiae	Lai	*L. interrogans*	Pathogenic	+	Brenner et al., 1999
5	Copenhageni	Icterohaemorrhagiae	M 20	*L. interrogans*	Pathogenic	+	Brenner et al., 1999
6	Muenchen	Australis	München C 90	*L. interrogans*	Pathogenic	+	Brenner et al., 1999
7	Pomona	Pomona	Pomona	*L. interrogans*	Pathogenic	+	Brenner et al., 1999
8	Pyrogenes	Pyrogenes	Salinem	*L. interrogans*	Pathogenic	+	Brenner et al., 1999
9	Icterohaemorrhagiae	Icterohaemorrhagiae	RGA	*L. interrogans*	Pathogenic	+	Brenner et al., 1999
10	Zhenkang	Javanica	L82	*L. borgpetersenii*	Pathogenic	+	Brenner et al., 1999
11	Sejroe	Sejroe	M84	*L. borgpetersenii*	Pathogenic	+	Brenner et al., 1999
12	Ballum	Ballum	Mus 127	*L. borgpetersenii*	Pathogenic	+	Brenner et al., 1999
13	Kenya	Ballum	Nijenga	*L. borgpetersenii*	Pathogenic	+	Brenner et al., 1999
14	Tarassovi	Tarassovi	Perepelicin	*L. borgpetersenii*	Pathogenic	+	Brenner et al., 1999
15	Poi	Javanica	Poi	*L. borgpetersenii*	Pathogenic	+	Brenner et al., 1999
16	Hardjo-bovis	Sejroe	Sponselee	*L. borgpetersenii*	Pathogenic	+	Brenner et al., 1999
17	Bim	Autumnalis	1051	*L. kirschneri*	Pathogenic	+	Brenner et al., 1999
18	Mozdok	Pomona	5621	*L. kirschneri*	Pathogenic	+	Brenner et al., 1999
19	Cynopteri	Cynopteri	3522 C	*L. kirschneri*	Pathogenic	+	Brenner et al., 1999
20	Grippotyphosa	Grippotyphosa	Moskva V	*L. kirschneri*	Pathogenic	+	Brenner et al., 1999
21	Ratnapura	Grippotyphosa	Wumalasena	*L. kirschneri*	Pathogenic	+	Brenner et al., 1999
22	Proechimys	Pomona	1161 U	*L. noguchii*	Pathogenic	+	Brenner et al., 1999
23	Panama	Panama	CZ 214 K	*L. noguchii*	Pathogenic	+	Brenner et al., 1999
24	Louisiana	Louisiana	LSU 1945	*L. noguchii*	Pathogenic	+	Brenner et al., 1999
25	Rushan	Australis	507	*L. noguchi*	Pathogenic	+	Brenner et al., 1999
26	Shermani	Shermani	1342 K	*L. santarosai*	Pathogenic	+	Brenner et al., 1999
27	Gorgas	Sejroe	1413 U	*L. santarosai*	Pathogenic	+	Brenner et al., 1999
28	Tropica	Pomona	CZ 299	*L. santarosai*	Pathogenic	+	Brenner et al., 1999
29	Bananal		Aa14	*L. santarosai*	Pathogenic	+	Brenner et al., 1999
30	Guaricura	Sejroe	Bov.G	*L. santarosai*	Pathogenic	+	Brenner et al., 1999
31	Manzhuang	Hebdomadis	A23	*L. alexanderi*	Pathogenic	+	Brenner et al., 1999
32	Mengla	Javanica	A85	*L. alexanderi*	Pathogenic	+	Brenner et al., 1999
33	Unipertama	Sejroe	K2-1	*L. weilii*	Pathogenic	+	Brenner et al., 1999
34	Langati	Tarassovi	M 39090	*L. weilii*	Pathogenic	+	Brenner et al., 1999
35	Mengdeng	Celledoni	M6906	*L. weilii*	Pathogenic	+	Brenner et al., 1999
36	Celledoni	Celledoni	Celledoni	*L. weilii*	Pathogenic	+	Brenner et al., 1999
37	Sarmin	Sarmin	Sarmin	*L. weilii*	Pathogenic	+	Brenner et al., 1999
38	Coxi	Javanica	Cox	*L. weilii*	Pathogenic	+	Brenner et al., 1999
39	Pingchang	Ranarum	80-412	*genomospecies 1*	Pathogenic	+	Brenner et al., 1999
40	Sofia	Javanica	Sofia 874	*L. meyeri*	Pathogenic	+	Brenner et al., 1999
41	Perameles	Mini	Bandicoot 343	*L. meyeri*	Pathogenic	+	Brenner et al., 1999
42	Ranarum	Ranarum	ICF	*L. meyeri*	Pathogenic	+	Brenner et al., 1999
43	Lincang	Manhao	L14	*L. inadai*	Intermediate	+	Brenner et al., 1999
44	Kaup	Tarassovi	LT 64-68	*L. inadai*	Intermediate	+	Brenner et al., 1999
45	Shermani	Aguaruna	MW 4	*L. inadai*	Intermediate	−	Brenner et al., 1999
46	Lyme	Lyme	10	*L. inadai*	Intermediate	−	Brenner et al., 1999
47			5399	*L. broomii*	Intermediate	−	Levett et al., 2006
48			L 065	*L. broomii*	Intermediate	−	Levett et al., 2006
49	Hurstbridge	Hurstbridge	BUT 6	*L. fainei*	Intermediate	−	Perolat et al, 1998
50	Varillal	Hurstbridge	VAR010	*L. licerasiae*	Intermediate	−	Matthias et al., 2008
51	Semaranga	Semaranga	Veldrat Semarang 173	*L. meyeri*	Non-pathogenic	−	Victoria et al., 2008
52	Holland	Holland	WaZ Holland	*genomospecies 3*	Non-pathogenic	−	Brenner et al., 1999
53	Saopaulo	Semaranga	Sao Paulo	*genomospecies 5*	Non-pathogenic	−	Brenner et al., 1999
54	Andamana	Andamana	CH11	*L. biflexa*	Non-pathogenic	−	Brenner et al., 1999
55	Patoc	Semaranga	Patoc I	*L. biflexa*	Non-pathogenic	−	Brenner et al., 1999
56	Codice	Codice	CDC	*L. wolbachii*	Non-pathogenic	−	Brenner et al., 1999

**Table 2:** Other micro-organisms used in this study.

No.	Species
1	*Acinetobacter calcoaceticus*
2	*Bartonella henselae*
3	*Bacillus subtilis*
4	*Bifidobacterium longum*
5	*Bordetella bronchiceptica*
6	*Borrelia burgdorferi*
7	*Brucella melitensis*
8	*Burkholderia cepacia*
9	*Campylobacter jejuni*
10	*Candida albicans*
11	*Candida dublinensis*
12	*Candida glabrata*
13	*Candida krusei*
14	*Candida parapsilosis*
15	*Corynebacterium diphteriae*
16	*Corynebacterium xerosis*
17	*Enterobacter aerogenes*
18	*Enterococcus faecalis*
19	*Enterococcus faecium*
20	*Escherichia coli*
21	*Helicobacter pylori*
22	*Klebsiella pneumoniae*
23	*Lactobacillus plantarum*
24	*Legionella pneumophila*
25	*Leishmania donovani*
26	*Leptonema illini*
27	*Listeria monocytogenes*
28	*Mycobacterium africanum*
29	*Mycobacterium bovis*
30	*Mycobacterium leprae*
31	*Mycobacterium tuberculosis*
32	*Neisseria gonorrhoeae*
33	*Pasteurella multocida*
34	*Plasmodium falciparum*
35	*Proteus mirabilis*
36	*Pseudomonas aeruginosa*
37	*Rickettsia akari*
38	*Salmonella enterica*
39	*Staphylococcus aureus*
40	*Streptococcus pneumoniae*
41	*Streptococcus sanguis*
42	*Trypanosoma cruzi*
43	*Toxoplasma gondii*
44	*Treponema pallidum*
45	*Turneriella parva*
46	*Yersinia enterocolitica*

The sequence of PCR primers was deduced from sequences of strains belonging to the species *L. interrogans*. Because genetic relatedness is used to differentiate *Leptospira* species, the primer annealing efficiency and hence the amplification efficiency might vary depending on the species from which the template DNA was isolated. Therefore, in addition to the homologous strain M 20, we determined the analytic sensitivities of the PCR with DNA from heterologous strains 1342 K and Sarmin. As listed in Table S1, the analytical sensitivity ranged from 1 to 50 copies depending on the strain and the biological materials, spiked with *Leptospira*.

### Robustness of the assay

The real-time PCR appeared to be highly robust. Varying concentrations of primers and MgCl_2_ as well as changing the annealing temperature, the incubation and the elongation time did not markedly affect the number of cycles in which the reaction becomes detectable (Ct) (data not shown).

### Diagnostic sensitivity and specificity

Clinical serum and blood samples from 26 confirmed positive (15 by culture and 11 by serology) and 107 negative patients were enrolled in the study (Fig. S2). Since clinical signs and symptoms were too varied to summarize, we used hospitalization and reference to the Intensive Care Unit (ICU) as criteria for severity of the diseases (Table S2). All patients were from The Netherlands; approximately 50% of the infections were acquired abroad, usually during vacation in South-East Asia. 92% of the patients were hospitalized and 45.8% attended the ICU, implying that the vast majority of patients were severely ill. The male: female ratio was 96∶4 (Table S2), which is a typical ratio for The Netherlands.

Based on culture as the gold standard, the real-time PCR had a diagnostic sensitivity (DSe) and diagnostic specificity (DSp) of 100% and 93%, respectively (Table 3). All 8 PCR positive samples that had a negative culture seroconverted later on, implying a higher actual DSp. When using confirmation by culture and serology as the gold standard, the DSe was lower (89%) as expected while the DSp was higher (100%). By grouping these PCR results on basis of the day of sample collection, i.e. 1–4 days and 5–10 days after onset of the disease, the DSe was 100% and 69%, respectively (Table 3). This supports a much higher value of the test at the early acute stage of the disease when bacterial loads are probably highest.

**Table 3:** The diagnostic sensitivity, specificity and confidence interval.*

Day of illness	Reference Standard	TP	FP	TN	FN	DSe (%)	CI (%)	DSp (%)	CI (%)
**up to 4**	Culture	9	3	63	0	100	63–100	96	86–99
**up to 4**	Culture + Serology	12	0	63	0	100	70–100	100	93–100
**5 to 10**	Culture	6	5	47	0	100	52–100	90	78–96
**5 to 10**	Culture + Serology	11	0	46	5	69	41–88	100	90–100
**1 to 10**	Culture	15	8	110	0	100	75–100	93	87–97
**1 to 10**	Culture + Serology	23	0	107	3	89	69–97	100	96–100

* TP, true positive; FP, false positive; TN, true negative; FN, false negative.

### Reproducibility and repeatability of the assay

All 20 positive tissue samples gave positive results and all 20 negative tissue samples were negative in each of the distinctly performed tests implying a perfect ‘analytical’ repeatability and reproducibility of the assay. Triplicate execution of the PCR on the clinical samples in fact provides an estimate of the practical repeatability. In 87.0% (20/23) of the samples two or more of the triplicates was positive and in 13% (3/23) one of the triplicates was positive (Fig. S2), implying that the diagnostic repeatability might be lower. This is probably due to low concentrations of leptospires in samples taken later in the acute phase because 85.0% of the scores of ≥2/3 triplicates were obtained in samples taken within the first 5 days of illness (data not shown).

## Discussion

Conventional diagnosis of leptospirosis mainly relies on serological techniques. These methods reach only suitable levels of sensitivity at a late acute phase of the disease when antibiotic treatment may be less effective. Leptospires are present in the blood during the first 5 to 10 days after onset of the disease. Direct detection of them would provide an excellent means to give early confirmation of clinical suspicion. Direct observation of leptospires in blood samples by darkfield microscopy is notoriously unreliable and not recommended [2]. Isolation of leptospires can take up to months and does not contribute to early diagnosis. Detection of leptospires through specific PCR amplification of its DNA has been championed as a promising alternative for two decades. Several conventional PCR tests for the specific detection of leptospiral DNA from body fluids and tissues have been described [8]–[11], [27], [28]. Unfortunately evaluation of the clinical applicability has only been done for two of such PCRs on a limited scale [13], [29], leaving the value of the conventional PCR for the laboratory diagnosis unclear. A further drawback of the conventional PCR is that the technique is particularly prone to contamination resulting to false positive outcomes [30]. It is not always clear whether serologically or culture negative but PCR positive samples reflect a higher sensitivity of the PCR or are due to contamination [31].

Real-time PCR, either using molecular beacons or SYBR Green technology has the advantage that it gives a result much quicker than conventional PCR and is less prone to contamination. By now, several real-time PCRs have been developed for the detection of leptospires [15], [16], [18], [19] but solid evaluations are lacking and therefore their usability remains uncertain. An auspicious exception is the recently described evaluation of a Taq-man real-time PCR [17]. This test showed an analytical sensitivity of 10 homologous genome copies and when compared with culture proven leptospirosis patients had a DSe and DSp of 100% and 93%, respectively.

Here we describe the development and evaluation of a real-time PCR based on the SYBR Green technology targeting the *secY* gene. The primer pair we selected showed high specificity for detection of pathogenic leptospires, excluding all saprophytic strains tested and the vast majority of intermediate ones. The lack of amplification of DNA from most intermediate strains is not unexpected because their *Leptospira* species form a separate intermediate clade situated between pathogenic and saprophytic species [23], [32]. This signifies a difference in DNA composition compared to pathogenic species apparently resulting in a too low annealing capacity of the primers hampering the amplification. In our opinion, this is of little relevance for the diagnostic potential of the test. Infection of patients with intermediate leptospires is a relatively rare event and their pathogenic status is as yet doubtful. Even though some of the intermediate *Leptospira* spp. have been described as clinical isolates, virulence cannot convincingly be demonstrated [33], [34]. No cross-reaction was found with other micro-organisms, which is an important feature because *secY* is a house-keeping gene that has been demonstrated in many prokaryotic species.

A major advantage of using the *secY* gene is its great phylogenetic potential [22], [24], [25]. We recently demonstrated that a small 245 bp segment of *secY*, flanked by the primer pair G1/G2 [11], had a high phylogenic power almost equaling that of the whole gene thus making it a feasible and interesting target for speciation by less sophisticated laboratories [23]. We found that the 201 bp fragment of the gene amplified in this real-time PCR assay had a similar phylogenetic potential as the 245 bp G1/G2 restricted fragment, making this target an attractive alternative for sequencing and phylogeny following amplification in a conventional format (Fig. S1).

The real-time PCR was validated using the specific instructions from OIE [35]. We found an analytical sensitivity of 1 to 50 copies, depending on the degree of homology between strains from different species and the type of sample used for extracting *Leptospira* DNA. Taking into account the DNA extraction and PCR protocols used in this study, detection of 1 genome copy per reaction equals a concentration of 100 leptospires per ml culture medium. This implicates a detection range from 100 to 5000 leptospires per ml or tissue equivalent, provided that the DNA extraction is efficient. Hence the high analytical sensitivity cannot be translated in a high practical efficiency, due to the sample processing step in which DNA is not concentrated. Our main future focus is therefore on developing a more adequate extraction procedure.

Notably DNA extracted from urine and kidney samples contained inhibitors. Both sample types are not essential for early diagnosis of leptospirosis but have value in other situations such as post-mortem investigations. We addressed the residual inhibition in two ways. For urine we introduced an extra washing step in the extraction procedure as most optimal tactic. For kidney samples preparing 1∶10 dilutions of the extracted DNA appeared the best approach. Both methods have the disadvantage of losing or diluting target DNA but overall the approaches led to markedly higher success rates. Inhibition is a real problem as this leads to false-negative results. To provide a tool to check on inhibitory effects in the PCR we introduced an IAC.

For early diagnosis, blood and serum are ideal samples. The immune system of the human body clears the bacteria from the blood after approximately 5–7 days after appearance of clinical manifestations. From one hand, the real-time PCR had a DSe of 100% when performed within the first four days of illness, which statistically represents a bias, as leptospires are still present at high concentrations in the patients' blood. On the other hand early confirmation of leptospirosis is of utmost importance for initiating adequate treatment. Therefore, from a clinical point of view, the high DSe at the early stage of illness signifies a great value for clinical decision making.

It should be noted that the very promising results of clinical evaluation in this study have been achieved with samples from Dutch patients. Currently half of the infections are acquired in The Netherlands where the serovars Copenhageni, Icterohaemorrhagiae and Grippotyphosa are dominant. This might induce a bias of the performance of the test. For this reason, the last stages of the OIE validation scheme include field studies at other laboratories to assess clinical sensitivity and specificity under different circumstances. We are currently aiming at the implementation and evaluation of the test in endemic areas with a variety of causative serovars.

In this study, culture and serology were considered as gold standard to estimate the clinical sensitivity and specificity in order to measure eventual bias of the results of high bacterial loads in culture and PCR positive samples alone. The overall sensitivity and specificity in this study were estimated as 93% and 100%, respectively. The assay showed complete reproducibility and repeatability as well as high level of robustness since changing in critical PCR parameters has no or slight influence on overall results.

Testing kidney, lung and liver from two early deceased patients as well as some rodent kidneys proved clearly the usefulness of the real-time PCR as an effective tool for the detection of *Leptospira* in the distinct tissues. This shows the applicability of real-time PCR as a suitable diagnostic tool on post-mortem samples, overcoming the failure to confirm leptospirosis of early deceased patients by serology.

## Materials and Methods

### Ethics statement

Procedures for collecting patients' data and use of clinical specimens for laboratory service improvement falls under the umbrella of the ‘National Coordination Infectious Disease Control’ (Landelijke Coördinatie Infectieziektebestrijding, LCI) [36], ‘Centre for Infectious Disease Control’ (Centrum Infectieziektebestrijding, Cib), which is a formal body of the Netherlands Ministry of Health and resides in the National Institute for Public Health and Environment (RIVM) in Bilthoven, The Netherlands and thus were conducted in compliance with the regulation, policies and principles of the Dutch Public Health Service Policy. The procedure includes the processing of anonymous data from patients upon receipt of a written informed consent.

### *Leptospira* strains and others organisms

Fifty-six *Leptospira* strains belonging to pathogenic, non-pathogenic and intermediate *Leptospira* spp. (Table 1) and 46 other micro-organisms (Table 2) were included in this study. *Leptospira* strains were from the collection of the WHO/FAO/OIE and National Leptospirosis Reference Centre in Amsterdam, The Netherlands. Other micro-organisms or their genomic DNA were gifts from colleagues from the Department of Biomedical Research and from other institutions.

### DNA extractions

Leptospires were propagated at 30°C in EMJH liquid media as described by Ellinghausen and McCullough [37] as modified by Johnson and Harris [38].

The number of bacteria per ml was determined by counting in a Helber bacteria chamber (Weber Scientific international, West Sussex BN15 8TN England) according to the standard protocol. All genomic DNA from leptospires and other micro-organisms in culture medium and from body fluids and tissues were extracted, purified and eluted in 0.1xTE buffer pH 8.0 by using the QIAamp DNA extraction kit (Qiagen, GmbH, D-40724 Hilden, Germany). This was done in accordance with the manufacturer's instructions with a slight modification for urine samples by adding one step of washing with the ALT buffer and proteinase K and spinning down the urine samples at 13000 rpm for two minutes instead of 8000 rpm. The quantity of *Leptospira* genomic DNA was estimated by measuring absorbance of DNA using the Spectrophotometer ND-1000 Nanodrop (3411 Silverside Rd, Bancroft Building, Wilmington, DE 19810, USA) and by visual comparison with Smart Ladder SF (Eurogentec S.A, Liege Science Park, 4102 Seraing, Belgium) after agarose gel electrophoresis. *Leptospira interrogans* serovar Copenhageni, strain M 20 was used as the basic strain for the development and initial evaluation of the real-time PCR. Based on the published genome of Copenhageni, one genome equivalent corresponds to 5.1 10^−15^ g DNA [39].

### Clinical samples

In this study we tested blood and serum samples from a consecutive cohort of 133 Dutch patients suspected of leptospirosis. Blood and serum samples from patients, clinically suspected for leptospirosis were submitted for confirmation to the WHO/FAO/OIE and National Leptospirosis Laboratory in Amsterdam in the period August 2005 till August 2008. This centre functions as the diagnostic centre for leptospirosis in The Netherlands and is accredited according to ISO 15189.

The following exclusion criteria have been applied (Fig. S2): (i) Patients from whom we did not receive a first sample within the first 10 days after onset of the disease; this was done because of the need for laboratory confirmation at the early acute phase. (ii) Patients from whom we received a first sample of <850 µl; this was done to guard the integrity of the standard procedure for diagnosis. (iii) No written informed consent for anonymous use of data available or expressed objections against the use of clinical specimens for improving laboratory services. All samples were investigated prospectively.

In addition, kidney, liver and lung tissue samples from two fatal, confirmed leptospirosis cases in the cohort with severe pulmonary haemorrhagic syndrome (SPHS) and Weil's syndrome were included in the study.

### Reference standards: Diagnostic culturing and serology

According to standard procedures of the reference centre, culturing was performed on first samples only. MAT and IgM ELISA were done on all samples included in the study.

A positive culture provides evidence of infection. For isolation, aliquots of 0.1 ml of serum or EDTA anticoagulated whole blood were inoculated into 6 ml EMJH culture medium and in Fletcher medium as described in text book literature [2]. Incubation was done at 30°C and cultures were inspected by darkfield microscopy for growth of leptospires at regular intervals up to 4 months.

The microscopic agglutination test (MAT), which is accepted as the standard reference test in the serodiagnosis of leptospirosis, was performed as per standard procedure [2], [40]. Performance of the IgM ELISA was as described [2], [34] using cut-off values defined in the diagnostic protocols in The Netherlands [36]. Seroconversion or a 4-fold or greater titer raise on paired samples was considered confirmative.

### Index test: the real-time PCR

#### Evaluation

Optimization and evaluation of the real-time PCR was done according to the protocol for validation of diagnostic PCRs of the OIE [35], which include working in separate clean rooms and the use of positive and negative controls. Real-time PCR was executed without knowledge on the outcome of the reference tests and vice versa. Tests were performed by skilled staff of the reference centre. For this paper, the instructions of Standards for the Reporting of Diagnostic accuracy testing (STARD) were followed [41].

#### Design of primers

*secY* sequences from pathogenic, intermediate and saprophytic *Leptospira* [23] were aligned to select primers that anneal efficiently with target DNA from pathogenic leptospires but not with that from intermediate en saprophytic species.

Real-time PCR standard parameters were taken into account in the design of the primers. The resulting primer set SecYIVF (5′-GCGATTCAGTTTAATCCTGC-3′) and SecYIV (5′-GAGTTAGAGCTCAAATCTA- AG-3′) are homologous to the *Leptospira* interrogans *S10-spc-α* locus (Genbank accession number AF115283) and amplify a 202 bp fragment between the locus positions 15744 and 15946. To determine their potential annealing specificity, the primer sequences were analysed with the BLAST search homology database [42].

#### Reaction conditions

Real-time PCRs were performed on an iQ^TM^5 Multicolour Real-Time PCR Detection System (Bio-Rad Laboratories, 2000 Alfred Nobel Drive, Hercules, CA94547 US) using the DNA-binding dye technique (SYBR Green). Unless otherwise stated the following reaction conditions were used: Reactions were performed in a total volume of 25 µl consisting of 1x iQ^TM^ SYBR Green Supermix (Bio-Rad) of 2x stock reagent containing 100 mM KCl, 40 mM Tris-HCI, pH 8.4, 0.4 mM of each dNTP, 50 units/ml iTaq DNA polymerase, 6 mM MgCl_2_, 20 nM fluoresein and stabilizers. Forward and reverse primers were added at a final concentration of 400 nM each. ICA was added in 0.5 µl volumes and DNA samples in 10 µl volumes. 10 µl sterile water was used instead of DNA template for negative controls. The amplification protocol consisted of 10 min at 95°C, followed by 40 cycles of amplification (95°C for 5 s, 54°C for 5 s, 72°C for 15 s). Subsequently, the reaction was stopped at 95°C for 2 minutes, cooled (20°C for 1 min) and melted (70–94°C with plate readings set at 0.5°C). The cut-off was set at Ct 35 that, in our hands, was the last cycle completely devoid of back-ground noise. To determine the optimal concentration of the PCR reagents and the optimal annealing temperature, different reagents concentrations were tested in combination with a temperature gradient and various incubation times. All experiments were repeated at least twice. The duration of the final PCR cycles was approximately 100 minutes including generation of the melting curve. The resulting data were analyzed using the software provided by the iQ5 system (Bio-Rad iQ^TM^5 2.0 Standard Edition Optical System Software, V2.0.148.060623).

#### Construction of internal control, IAC

In order to determine the inhibition in the biological samples and to identify false negative results an IAC was constructed according to Abdulmawjood et, al. [43], using the sequence of the *secY* gene of *Treponema pallidum*, strain Nichols (GenBank accession number AE000520) as a template. We selected a gene segment which yielded a longer amplicon size (249 bp) and higher Tm (86°C) than the *Leptospira* target in the real-time PCR. Primer pair secyicF 5′-GCGATTCAGTTTAATCCTGCCCGC- CTGGTACTTCCCGG-3′ and secyicR 5′-GAGTTAGAGCTCAAATCTAAGGCCACGCCCTCCCAACC-3′, consisted of a 3′ part homologous to the *T. pallidum* sequence and a 5′ sequence corresponding to the primers SecYIVF and SecYIV, respectively. The IAC amplicon was constructed by performing a conventional PCR as follows; enzyme activation for 10 minutes at 95°C followed by 34 cycles of denaturation for 30 seconds at 95°C, annealing for 30 seconds at 50°C and elongation for 30 seconds at 72°C and a final extension step for 7 minutes at 72°C. The PCR product was purified using MinElute PCR Purification Kit as per manufacturer's recommendation (Qiagen, Germany) and cloned into the vector pGEM-T (BaseClear Group, Leiden, The Netherlands) according to standard procedures. To identify the optimal concentration for use of this IAC in the real-time PCR, 0.5 µl aliquots of serial 10-fold dilutions of IAC ranging from undiluted till a 10^12^ fold dilution were tested in the presence of 1000 genome copies of DNA from strain M 20 per reaction. The optimal IAC concentration was established on the criterion that a reliable IAC amplicon was always present in the *Leptospira* negative samples while no or a faint IAC product was generated in *Leptospira* positive samples.

### Analytical specificity and sensitivity

To investigate whether the deduced primer set SecYIVF and SecYIV specifically amplify DNA from pathogenic *Leptospira*, we tested 5.1 10^−12^ g DNA per reaction from each of 42 *Leptospira* strains belonging to eight pathogenic species and from 14 strains of nine intermediate and saprophytic species (Table 1) as well as 46 other clinical important micro-organism (Table 2).

To estimate the detection threshold of the PCR (analytical sensitivity), a standard curve was constructed using 10-fold serial dilutions DNA template of homologous strain M 20 and heterologous strains 1342 K and Sarmin producing relatively intermediate and weak amplification, respectively, under these standardized conditions. For fine-tuning of the end-point dilution, the last positive 10-fold dilutions still giving a product were subjected to subsequent 2-fold serial dilutions, performed independently by two different persons. According to the OIE recommendation [35], we set the end-point at the dilution in which the assay could not detect the target in at least 5% of the replicates.

To assess the extent of potentially inhibiting effects of biological materials such as serum, blood, urine and tissue on the analytical sensitivity, 10-fold serial dilutions followed by 2-fold serial dilution of biological materials spiked with strains M 20, 1342 K and Sarmin were targeted by the assay.

### Robustness

To explore the effect of changing critical PCR parameters, PCRs were performed with annealing temperature ranging from 52.5°C to 55.1°C and annealing time of 10, 15 and 20 seconds. To determine the influence of changing the concentration of MgCl_2_ and primers, these were tested in the ranges of 3.0–4.0 mM and 0.2–0.4 µM, respectively. All the experiments were done in triplicate and repeated at least twice.

### Repeatability and reproducibility

The degree of agreement between replicates within the same run (repeatability) or between replicates tested by different persons (reproducibility) was measured based on the OIE recommendation [35]. We tested blinded kidney samples from 20 confirmed positive and 20 negative rodents, starting from DNA extraction, by two different persons in triplicate for at least two times.

### Diagnostic sensitivity (DSe) and specificity (DSp)

In first instance, we used positive cultures to corroborate the infection for estimating DSe and DSp [35]. To assess an eventual bias of PCR positive results towards high bacterial loads in culture positive samples, we additionally performed calculations of DSe and DSp on basis of reference standard, i.e. positive culture and or serology.

Real-time PCR was executed in triplicate. Samples with two or more positive reactions were scored as PCR positive. For confirmation, samples with a single positive reaction were repeated in triplicate. Samples were included when at least one of the triplicates was again positive (Fig. S2). Sensitivity, specificity and confidence intervals were calculated according to standard literature [44]–[46].

### Phylogeny

DNA sequence clustal alignments were done using Vector NTI 10 software (Invitrogen).

Phylogenetic analysis was conducted using MEGA4 [47]. One thousand bootstrap replications were used to provide confidence in the nodes. The tree was constructed by the Neighbor-Joining method using the Jukes-Cantor model [47].

## Supporting Information

Figure S1 Circular phylogenetic trees elaborated using the Neighbor-joining method. Phylogenetic tree deduced from SecYIV-IVF (A) and G1-G2 (B) restricted sequences using 1000 bootstrapping replicates.
(0.54 MB TIF)

Figure S2 Inclusion flow chart. Flow diagram showing inclusion of index patients and outcomes of the reference and index tests.
(0.46 MB TIF)

Table S1 Analytical sensitivities*. * Detection threshold; Numbers of copies detected in one reaction.
(0.02 MB DOC)

Table S2 Index patients. Information of index patients. ICU, intensive care unit; N, no; Y, yes; U, unknown; -, negative; +, positive
(0.05 MB DOC)
