# Supplementary figures and images for: Development and Validation of a Real-Time PCR for Detection of Pathogenic Leptospira Species in Clinical Materials
Source: PLoS One. 2009 Sep 18;4(9):e7093. doi: 10.1371/journal.pone.0007093 (PMC2740861; doi:10.1371/journal.pone.0007093)

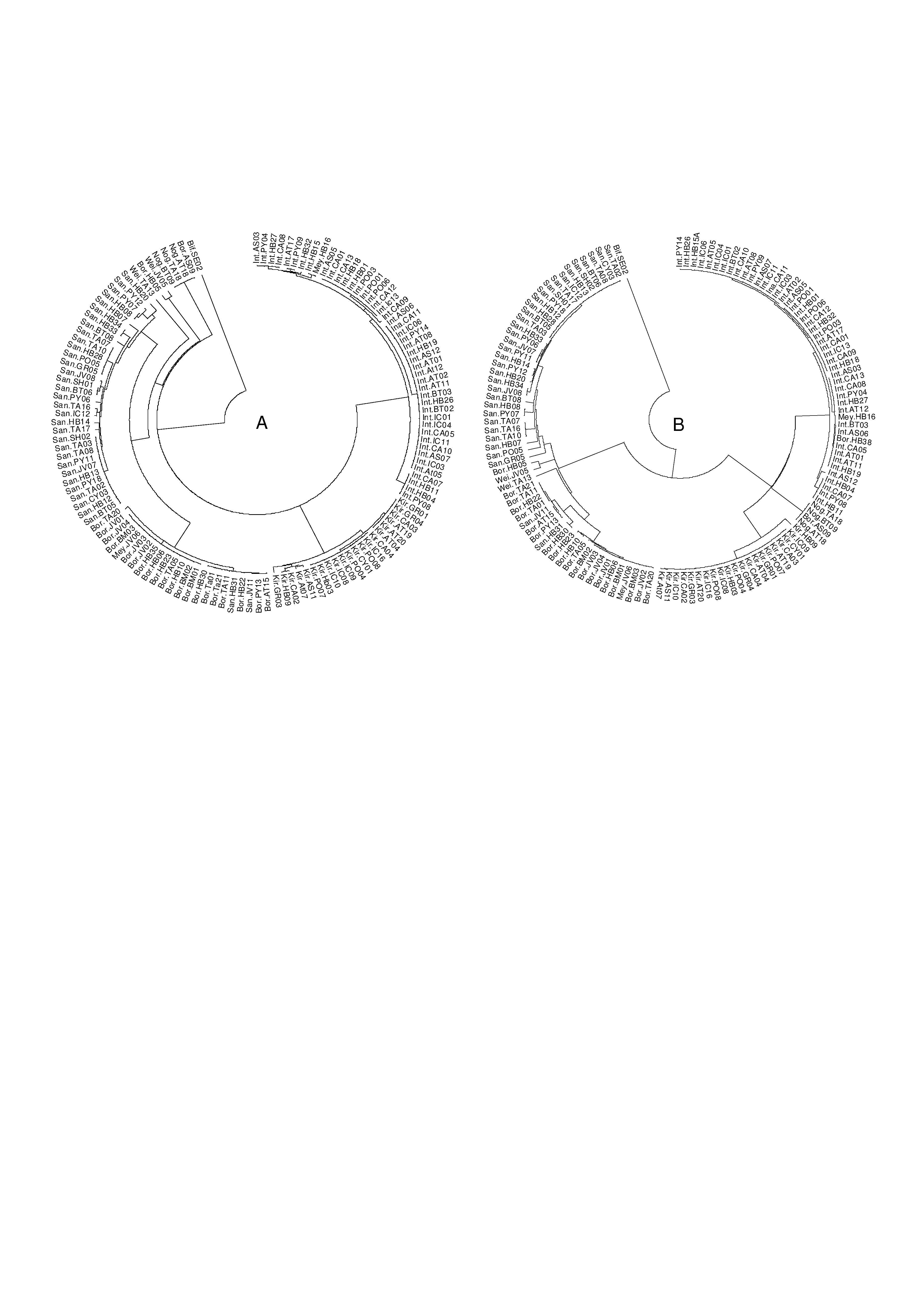

Supplement: Figure S1 — Circular phylogenetic trees elaborated using the Neighbor-joining method. Phylogenetic tree deduced from SecYIV-IVF (A) and G1-G2 (B) restricted sequences using 1000 bootstrapping replicates. (0.54 MB TIF) [file pone.0007093.s001.tif]

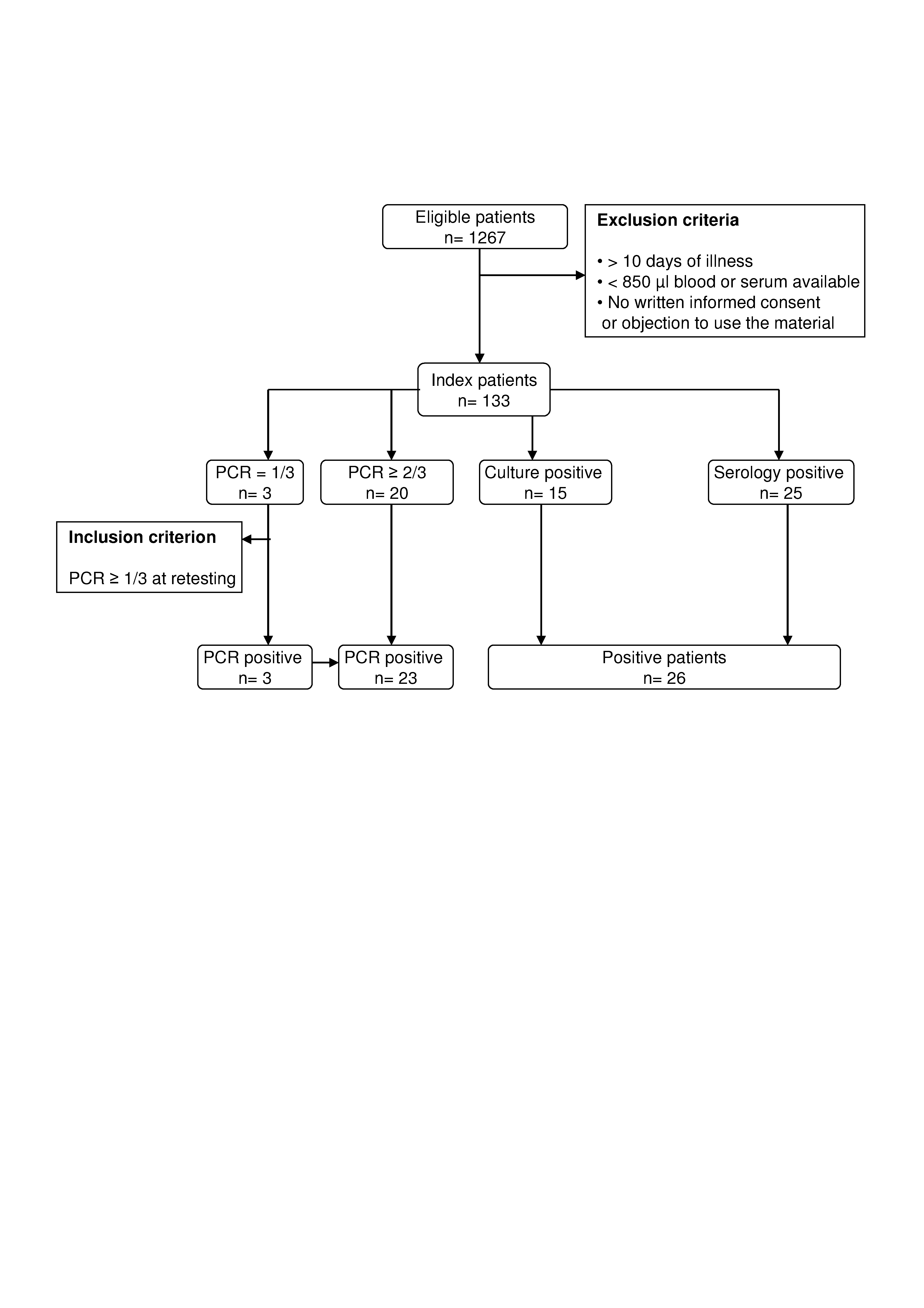

Supplement: Figure S2 — Inclusion flow chart. Flow diagram showing inclusion of index patients and outcomes of the reference and index tests. (0.46 MB TIF) [file pone.0007093.s002.tif]
