# Supplementary material for: Development and Validation of a Real-Time PCR for Detection of Pathogenic Leptospira Species in Clinical Materials
Source: PLoS One. 2009 Sep 18;4(9):e7093. doi: 10.1371/journal.pone.0007093 (PMC2740861; doi:10.1371/journal.pone.0007093)
Supplement: Table S1 — Analytical sensitivities*. * Detection threshold; Numbers of copies detected in one reaction. (0.02 MB DOC) [file pone.0007093.s003.doc]

**Table S1. Analytical sensitivities***

| **Strain** | **Culture** | **Spiked serum** | **Spiked blood** | **Spiked kidney** |
| --- | --- | --- | --- | --- |
| **M 20** | 1.0 | 10 | 20 | 20 |
| **1342 K** | 1.2 | 15 | 30 | 30 |
| **Sarmin** | 1.5 | 30 | 50 | 50 |

* Detection threshold; Numbers of copies detected in one reaction.
