# Supplementary material for: Development and Validation of a Real-Time PCR for Detection of Pathogenic Leptospira Species in Clinical Materials
Source: PLoS One. 2009 Sep 18;4(9):e7093. doi: 10.1371/journal.pone.0007093 (PMC2740861; doi:10.1371/journal.pone.0007093)
Supplement: Table S2 — Index patients. Information of index patients. ICU, intensive care unit; N, no; Y, yes; U, unknown; -, negative; +, positive (0.05 MB DOC) [file pone.0007093.s004.doc]

**Table S2.** Index patients

| **Patient** | **Gender** | **Age** | **Infected In The Netherlands** | **Hospitalization** | **ICU** | **Culture** | **Serology** | **PCR** |
| --- | --- | --- | --- | --- | --- | --- | --- | --- |
| 1 | Male | 56 | N | Y | Y | + | + | + |
| 2 | Female | 54 | U | Y | N | + | + | + |
| 3 | Male | 61 | Y | Y | U | - | + | + |
| 4 | Male | 55 | Y | Y | U | + | + | + |
| 5 | Male | 59 | Y | Y | Y | - | + | - |
| 6 | Male | 32 | N | N | N | + | - | + |
| 7 | Male | 45 | Y | Y | Y | + | + | + |
| 8 | Male | 39 | Y | Y | N | + | + | + |
| 9 | Male | 83 | Y | Y | Y | - | + | + |
| 10 | Male | 48 | Y | Y | N | - | + | + |
| 11 | Male | 54 | N | Y | N | - | + | + |
| 12 | Male | 41 | Y | Y | Y | + | + | + |
| 13 | Male | 25 | Y | Y | N | + | + | + |
| 14 | Male | 48 | N | Y | N | - | + | + |
| 15 | Male | 30 | N | Y | Y | + | + | + |
| 16 | Male | 34 | N | U | U | + | + | + |
| 17 | Male | 50 | Y | Y | Y | - | + | + |
| 18 | Male | 29 | N | Y | N | - | + | - |
| 19 | Male | 28 | N | N | N | + | + | + |
| 20 | Male | 51 | N | Y | N | + | + | + |
| 21 | Male | 57 | Y | Y | Y | + | - | + |
| 22 | Male | 75 | Y | Y | Y | - | + | + |
| 23 | Male | 15 | Y | Y | Y | + | + | + |
| 24 | Male | 41 | N | Y | N | + | + | + |
| 25 | Male | 28 | Y | Y | Y | - | + | + |
| 26 | Male | 22 | N | Y | N | - | + | - |

Information of index patients. ICU, intensive care unit; N, no; Y, yes; U, unknown; -, negative; +, positive.
